# Self-control Predicts Exercise Behavior by Force of Habit, a Conceptual Replication of Adriaanse et al. (2014)

**DOI:** 10.3389/fpsyg.2017.00190

**Published:** 2017-02-13

**Authors:** Marleen Gillebaart, Marieke A. Adriaanse

**Affiliations:** Self-regulation Lab, Social, Health, and Organizational Psychology, Utrecht UniversityUtrecht, Netherlands

**Keywords:** exercise, health, behavior, trait self-control, habits

## Abstract

A recent study suggests that habits play a mediating role in the association between trait self-control and eating behavior, supporting a notion of effortless processes in trait self-control (Adriaanse et al., 2014). We conceptually replicated this research in the area of exercise behavior, hypothesizing that these associations would generalize to other self-control related behaviors. Sufficient exercise is essential for several health and well-being outcomes, and therefore many people intend to exercise. However, the majority of the population does not actually exercise to a sufficient or intended extent, due to competing temptations and short-term goals. This conflict makes exercise a typical example of a self-control dilemma. A within-subjects survey study was conducted to test associations between trait self-control, habit strength, and exercise behavior. Participants were recruited at a local gym. Results demonstrated that trait self-control predicted exercise behavior. Mediation analysis revealed that the association between self-control and exercise was mediated by stronger exercise habits, replicating findings by Adriaanse et al. (2014). These results highlight the relevance of self-control in the domain of exercise. In addition, they add to a growing body of knowledge on the underlying mechanisms of trait self-control on behavior that point to habit—rather than effortful impulse inhibition—as a potential key to self-control success.

## Introduction

Self-control is a key personality trait because it is associated with a myriad of positive life outcomes (e.g., Tangney et al., 2004), including happiness (Cheung et al., 2014; Hofmann et al., 2014) and health (Moffitt et al., 2011). Self-control has been the topic of many studies in various domains. More recently, studies have moved beyond demonstrating positive effects of self-control to examining the underpinnings of this essential trait. To provide further support for this novel account of self-control, in the current paper, we report a conceptual replication of a recent study showing that habit strength underlies the association between trait self-control and behavior (Adriaanse et al., 2014), in order to strengthen this novel account of self-control.

Self-control has often been defined as an effortful process that requires resources and an “active self.” Indeed, the classic “strength model of self-control” describes self-control as drawing from a self-regulatory resource that is susceptible to depletion and subsequent self-control failure (Baumeister et al., 1998, 2007). However, a recent shift in the conceptualization of self-control has produced novel perspectives on *effortless* pathways to successful self-control that are especially relevant to the trait aspect of self-control. In contrast to the classic view of self-control as the effortful inhibition to impulses (Baumeister et al., 2007), a meta-analysis revealed that trait self-control is actually more important in *habitual* compared to *deliberate* behaviors (De Ridder et al., 2012). These findings suggest that there are also pathways to successful self-control that are not based on effortful inhibition, such as for instance adaptive habits (Gillebaart and De Ridder, 2015).

Habits are characterized by their automaticity (Verplanken and Aarts, 1999; Lally et al., 2011). Because of this automaticity, executing habitual behaviors does not require conscious intent or effort (Aarts et al., 1998; Gardner et al., 2012). At first glance, trait self-control and habits may seem at odds with each other as one is characterized by effortless behavior (habit) and the other is generally related to effortful behavior (self-control). This apparent paradox may be resolved by recent insights from trait self-control research, which suggest that rather than being opposite forces, habits and self-control are actually strongly related predictors of behavior. This effortless self-control notion has been supported by a recent study showing that habit strength mediates the relation between trait self-control and snack intake (Adriaanse et al., 2014) and a set of studies revealing associations between self-control and habits (Galla and Duckworth, 2015).

These studies suggest that habits—rather than being an opposite force—are in fact an underlying mechanism of self-control. This result implies that people with high levels of trait self-control actually may not be successful at achieving their long-term self-control related goals because they effortfully inhibit impulsive behaviors, but rather because they can rely on effortless habitual behaviors. Counter intuitively, this may in fact mean that people with higher levels of self-control do not need to “use” their self-control as often as people with lower levels of trait self-control who do not have these habits to lean on. In line with this, Hofmann et al. (2012) demonstrated that people with higher trait self-control report fewer problematic temptations in their environment. Furthermore, Gillebaart et al. (2016) found that people with higher trait self-control experience weaker experienced conflict between short-term and long-term goals. As Gillebaart and De Ridder (2015) suggest, this may be because adaptive habits allow people with higher trait self-control to go about their lives in a way that allows them to routinely avoid problematic situations, and make the “right” choices in line with their long-term goals. Habits as an underlying process in trait self-control may therefore explain why people high in trait self-control are successful in achieving their long-term goals.

In the original study by Adriaanse et al. (2014), snacking behavior was assessed, since healthy eating represents a typical self-control dilemma. Self-control dilemmas are situations in which long-term goals clash with short-term goals, and self-control is required to steer behavior toward these long-term goals and away from the short-term, often hedonic based goals (Friese et al., 2011). Besides healthy eating, exercise is also considered a prototypical self-control dilemma: while it is supportive of a number of long-term goals such as health and fitness, it often competes with short-term goals, such as watching one's favorite TV show, or staying in bed a little longer (Giner-Sorolla, 2001). Exercising on a regular basis benefits physical as well as mental health (World Health Organization, 2015). For instance, it promotes muscular and skeletal strength, as well as mood and mental health, while a lack of exercise is associated with health issues such as obesity (Ross et al., 2000), and cardiovascular disease (Williams, 2001). In view of its health benefits, many people intend to exercise on a regular basis (Norcross et al., 2002). However, the majority of the adult population does not exercise to a sufficient extent (Dishman and Buckworth, 2001). This may be due to self-control issues: high levels of self-control lead to healthier exercise behavior (Hagger et al., 2009), and an array of research has demonstrated self-control's importance for exercise activities like basketball shooting and dart throwing (Englert and Bertrams, 2012), and for adoption and maintenance of exercise programs (Sniehotta et al., 2005; Wills et al., 2007).

We conducted a conceptual replication of Adriaanse's et al. (2014) study in order to strengthen the recently surfaced, novel perspective on trait self-control as being, at least partially, driven by automatic processes like habits. Exercise behavior was the dependent variable in the current study. More specifically, a field study was conducted among gym members from a local gym to test the hypothesis that trait self-control level positively predicts how much people take part in exercise behavior, with exercise habit strength mediating this association.

## Methods

### Participants

One hundred and thirty-four participants were recruited from a local gym's database. The sample consisted of 52 males and 82 females, with a mean age of 37.93 years (*SD* = 12.44, range 18–65). Participants were enrolled in a lottery for a wellness voucher worth 25 euro. The study was conducted in line with ethical standards described by the Medical Research Involving Human Subjects Act (WMO, 2012). This Act exempts research on healthy human subjects from ethical review provided it does not involve any invasion of participants' integrity. According to national guidelines our procedure did not invade participants' integrity, and was hence not subject to the WMO. Informed consent was obtained from each participant prior to participation.

### Procedure

Participants were approached via email, and asked to fill out an online survey. Demographic data was collected, as well as questionnaires about their self-control, exercise habit strength, and exercise behavior over the past month and week. Afterwards, participants were debriefed.

### Materials

Trait self-control was measured using the Brief Trait Self-Control Scale (Tangney et al., 2004). This scale consists of 13 statements (e.g., “I am good at resisting temptations”), of which nine are recoded to higher scores reflecting higher self-control. Statements were scored on 5-point Likert scales (1—not at all applicable to me, 5—very much applicable to me). The scale proved reliable with a Cronbach's α of 0.81.

Exercise habit strength was measured with the Self-Report Habit Index (SRHI; Verplanken and Orbell, 2003). The SRHI taps into three components of habits: experience of repetition (three items, e.g., “Exercising is something I have been doing for a long time”), automaticity (seven items, e.g., “Exercising is something I do without thinking”), and expression of identity (two items, e.g., “Exercising is something that is typically ‘me”’). Statements were scored on 7-point Likert scales (1—totally disagree, 7—totally agree). The scale proved reliable with a Cronbach's α of 0.97.

The dependent variable exercise behavior was assessed with two questions. One question assessed how many times per week participants reported to have exercised in the 3 months preceding the day of the study. Exercise was defined as a minimum of 20 min intense physical exercise (Haskell et al., 2007), and participants were provided with examples of such exercise. Participants could indicate that they exercised less than once a week, once a week, or twice a week or more in the preceding 3 months. The second question assessing exercise behavior comprised the number of minutes participants reported they had exercised in the week preceding the day of the study. Additional measures were used for educational purposes. These measures comprised a goal framing measure and a motivation measure, both of which are beyond the scope of this study.

## Results

Participants in the sample had a mean Body Mass Index (BMI) of 23.99 (*SD* = 3.49). Mean trait self-control level was 3.31 (*SD* = 0.57), and mean exercise habit strength was 4.33 (*SD* = 1.83). On average, participants reported exercising for 159.79 (*SD* = 145.62) minutes in the week before the study. Of the participants, 24 indicated to have exercised less than once a week, 16 to have exercised once a week, and 86 to have exercised twice a week or more in the preceding 3 months.

Spearman's rank-order correlations and Pearson product-moment correlations were computed to assess associations of trait self-control and habit strength with exercise behavior. All correlations are presented in Table 1. Trait self-control was significantly and positively related to minutes of exercise minutes in the preceding week (*r* = 0.33, *p* < 0.001) as well as to times exercised per week in the preceding 3 months (*r*_*p*_ = 0.18, *p* = 0.045). Trait self-control was also significantly and positively related to strength of exercise habit (*r* = 0.31, *p* < 0.001). Strength of exercise habit and actual minutes of exercise were likewise significantly and positively correlated (*r* = 0.56, *p* < 0.001), as were strength of exercise habit and number of times exercised (*r*_*p*_ = 0.68, *p* < 0.001). Age and BMI were not correlated to any of the key variables.

**Table 1:** **Correlations between all variables, with degrees of freedom in parentheses following each correlation**.

	**1. Trait self-control**	**2. Self-report habit index**	**3. Minutes of exercise**	**4. Frequency of exercise**	**5. Age**	**6. BMI**
1.	−	0.31^**^ (130)	0.33^**^ (114)	0.18^*^ (124)	0.09 (130)	−0.17 (125)
2.	0.31^**^ (130)	−	0.56^**^ (114)	0.68^**^ (124)	−0.04 (130)	0.03 (125)
3.	0.33^**^ (114)	0.56^**^ (114)	−	0.70^**^ (114)	−0.09 (114)	−0.08 (110)
4.	0.18^*^ (124)	0.68^**^ (124)	0.70^**^ (114)	−	−0.01 (124)	0.06 (110)
5.	0.09 (130)	−0.04 (130)	−0.09 (114)	−0.01 (124)	−	0.36^**^ (127)
6.	−0.17 (125)	0.03 (125)	−0.08 (110)	0.06 (110)	0.36^**^ (127)	−

Correlations accompanied by

* are significant at the α = 0.05 level. Correlations accompanied by

** *are significant at the α = 0.001 level*.

The significant correlations between trait self-control, strength of exercise habit, and exercise behavior warranted mediation analyses. In this analysis, we tested whether the association between self-control level and exercise behavior was mediated by habit strength. Using Hayes (2012) PROCESS macro, the indirect effect between trait self-control and minutes of exercise in the preceding week through exercise habit strength was estimated at 45.56, with 5,000 bootstrapping samples. The 95% confidence interval did not include zero (95% CI [24.94, 75.71]), meaning the mediational pathway was significant. A similar mediation analysis demonstrated that the indirect effect between trait self-control and number of times exercised per week in the preceding 3 months through exercise habit strength was estimated at 0.36, with 5,000 bootstrapping samples. The 95% confidence interval did not include zero (95% CI [0.19, 0.56]), meaning that this mediational pathway was also significant. A higher level of exercise habit strength mediated the relationship between trait self-control and both measures of exercise behavior, in line with our hypothesis. A summary of the regression analysis is displayed in Table 2.

**Table 2:** **Summary table of regression analyses testing the mediation of habit strength in the association between self-control and exercise behavior**.

**MODEL SUMMARY**						
	***R*****^2^**	***MSE***	***F***	***P***		
Exercise frequency	0.52^*^	0.31	66.94	> 0.001		
Minutes of exercise	0.33^*^	14,440.25	27.93	> 0.001		
**DIRECT EFFECT OF SELF-CONTROL**						
	**Effect**	***SE***	***t***	***P***	**LLCI**	**ULCI**
Exercise frequency	−0.11	0.09	−1.20	0.23	−0.30	0.07
Minutes of exercise	0.41	21.39	1.91	0.06	−1.48	84.08
**INDIRECT EFFECT OF SELF-CONTROL**						
	**Effect**	***SE***	***t***	***p***	**LLCI**	**ULCI**
Exercise frequency	0.36^a^	0.09	−	−	0.19	0.56
Minutes of exercise	45.56^a^	12.74	−	−	24.94	75.71

*Self-control was added to the model as the independent variable, and self-reported habit strength as the mediator*.

Values accompanied by

* are significant at the α = 0.05 level. Effects accompanied by

a *are significant since the corresponding confidence interval does not include zero*.

The mediation results did not change when removing participants who indicated there were medical conditions influencing their ability to exercise (*n* = 19) from the sample. Removing the frequency item from the exercise habit strength index also did not change the results, indicating that the results reflect mediation by the automaticity aspect of habits (Verplanken, 2006).

## Discussion

The results from this field study were in favor of our hypotheses, suggesting that trait self-control positively predicted exercise behavior, and that this association was mediated by stronger exercise habits for people with a higher trait self-control level. Therefore, findings from this study conceptually replicated earlier findings by Adriaanse et al. (2014), who demonstrated similar associations between trait self-control, habit strength, and snacking behavior.

Both self-control and habits have frequently been mentioned as determinants for exercise behavior (Aarts et al., 1998; Sniehotta et al., 2005; Hagger et al., 2009; Phillips and Gardner, 2016). However, these different lines of research have not been unified until now. One reason for the lack of unification may be that, at first glance, these two predictors seem to be at odds with each other: self-control is traditionally believed to require effort, intent, and resources (Baumeister et al., 1998), while habits operate effortlessly, without that intent, and without depleting resources (Verplanken and Aarts, 1999; Orbell and Verplanken, 2010). Results from the current study demonstrate that in fact, habits, and trait self-control are very much related, which is in line with recent developments in research on self-control processes. Self-control comprises effortful inhibition of impulses but also seems to operate on an automatic level (De Ridder et al., 2012), and people with higher levels of self-control seem to benefit from adaptive routines rather than having to use effort and resources for each self-control dilemma (Adriaanse et al., 2014; Gillebaart and De Ridder, 2015). The current study demonstrates a first translation of this notion to exercise behavior.

The fact that engaging in exercise behavior involves effort is intuitively appealing. People often have to fit a healthy amount of exercise into their busy lives in order to reach a long-term goal of health and fitness, and need to steer themselves away from hedonic, short-term rewarding behaviors like being sedentary or sleeping in. But, if exercise behavior would completely depend on this effortful self-control process, this would paint a pretty hopeless picture of exercise initiation and maintenance, as self-control can be compromised due to for example previous exertions of self-control (Baumeister et al., 1998, 2007). Fortunately, the reality is less grim: people who go to the gym (or have an active gym membership), like our sample, actually do seem to be successful at working toward their exercise goal. Our results show that although their exercise behavior is predicted by their self-control level, they may use a different, less effortful self-control strategy when it comes to exercising: habits.

There are limitations to the current study that urge some caution when interpreting the results. A first limitation concerns the potential for establishing causality of the proposed relationships. Both on a statistical and conceptual level, caution needs to be applied when interpreting the causality of the reported results. Statistically, a first limitation pertains to the correlational nature of the design. Since none of the variables were manipulated in a controlled setting, causality of the proposed associations cannot be established. Moreover, mediation analysis is suited for testing significance of a mediational relationship, but not for identifying a mediator. This means that a significant mediation cannot rule out other causal models describing the correlational associations (Fiedler et al., 2011). Furthermore, the exercise behavior that participants reported on took place in a time period of 3 months before they filled in the survey. Strictly, this means that our predictors trait self-control and habit strength were measured at a later time than the behavior we wanted to predict with our mediation analysis. This could cause a problem for mediation analyses (MacKinnon and Fairchild, 2009). However, trait self-control as measured in this study is considered a stable personality trait, not susceptible to large fluctuations due to situational factors (Tangney et al., 2004). Moreover, there is an important advantage of using a design with retrospective reporting of exercise behavior: participants did not get the opportunity to change their behavior as a result of monitoring that could occur after filling in questions about their exercise behavior (Verhoeven et al., 2014), which we feel outweighs the limitations that follow from this design. Of course, an inherent limitation of self-report measures is that they are always to some extent subjective.

Conceptually, one could assume overlap between trait self-control, habit strength, and exercise behavior. However, the reported correlations between the variables are of a medium effect size, suggesting that the trait self-control, habit strength, and exercise behavior do in fact represent different concepts that are mutually associated. Taken together, as our findings strongly align with previous research (e.g., Adriaanse et al., 2014; Galla and Duckworth, 2015) we are relatively confident in our conclusion that self-control affects habit strength, which in turn influences exercise behavior.

This conceptual replication study has provided further insight into the underpinnings of successful self-control. The key to successfully keeping up a healthy eating or exercise routine may lie in the strength of the habit that is formed as a consequence of trait self-control. This implies that people who struggle with low levels of trait self-control could benefit from interventions based on habit formation to be able to exercise as effortlessly as their high trait self-control counterparts.

## Author contributions

MG and MA both contributed significantly to the research and manuscript.

### Conflict of interest statement

The authors declare that the research was conducted in the absence of any commercial or financial relationships that could be construed as a potential conflict of interest.

## References

[B1] Aarts H. Verplanken B. Van Knippenberg A. (1998). Predicting behavior from actions in the past: repeated decision making or a matter of habit? J. Appl. Soc. Psychol. 28, 1355–1374. 10.1111/j.1559-1816.1998.tb01681.x

[B2] Adriaanse M. A. Kroese F. M. Gillebaart M. De Ridder D. T. D. (2014). Effortless inhibition: habit mediates the relation between self-control and unhealthy snack consumption. Front. Psychol. 5:444. 10.3389/fpsyg.2014.00444 24904463 PMC4032877

[B3] Baumeister R. F. Bratslavksy E. Muraven M. Tice D. M. (1998). Ego depletion: is the active self a limited resource? J. Pers. Soc. Psychol. 74, 1252–1265. 10.1037/0022-3514.74.5.1252 9599441

[B4] Baumeister R. F. Vohs K. D. Tice D. M. (2007). The strength model of self-control. Curr. Dir. Psychol. Sci. 16, 351–355. 10.1111/j.1467-8721.2007.00534.x

[B5] Cheung T. T. Gillebaart M. Kroese F. De Ridder D. (2014). Why are people with high self-control happier? The effect of trait self-control on happiness as mediated by regulatory focus. Front. Psychol. 5:722. 10.3389/fpsyg.2014.00722 25071683 PMC4085873

[B6] De Ridder D. T. D. Lensvelt-Mulders G. Finkenauer C. Stok F. M. Baumeister R. F. (2012). Taking stock of self-control: a meta-analysis of how trait self-control relates to a wide range of behaviors. Person. Soc. Psychol. Rev. 16, 76–99. 10.1177/1088868311418749 21878607

[B7] Dishman R. K. Buckworth J. (2001). Exercise Psychology. Champaign, IL: Human Kinetics.

[B8] Englert C. Bertrams A. (2012). Anxiety, ego depletion, and sports performance. J. Sports Exerc. 34, 580–599. 10.1123/jsep.34.5.580 23027229

[B9] Fiedler K. Schott M. Meiser T. (2011). What mediation analysis can (not) do. J. Exp. Soc. Psychol. 47, 1231–1236. 10.1016/j.jesp.2011.05.007

[B10] Friese M. Hofmann W. Wiers R. (2011). On taming horses and strengthening riders: recent developments in research on interventions to improve self-control in health behaviors. Self Identity 10, 336–351. 10.1080/15298868.2010.536417

[B11] Galla B. M. Duckworth A. L. (2015). More than resisting temptation: beneficial habits mediate the relationship between self-control and positive life outcomes. J. Pers. Soc. Psychol. 109, 508–525. 10.1037/pspp0000026 25643222 PMC4731333

[B12] Gardner B. Lally P. Wardle J. (2012). Making health habitual: the psychology of ‘habit-formation’ and general practice. Br. J. Gen. Pract. 62, 664–666. 10.3399/bjgp12X659466 23211256 PMC3505409

[B13] Gillebaart M. De Ridder D. T. D. (2015). Effortless self-control: a novel perspective on response conflict strategies in trait self-control. Soc. Person. Psychol. Compass 9, 88–99. 10.1111/spc3.12160

[B14] Gillebaart M. Schneider I. K. De Ridder D. T. (2016). Effects of trait self control on response conflict about healthy and unhealthy food. J. Pers. 84, 789–798. 10.1111/jopy.12219 26269964

[B15] Giner-Sorolla R. (2001). Guilty pleasures and grim necessities: affective attitudes in dilemmas of self-control. J. Pers. Soc. Psychol. 80, 206–221. 10.1037/0022-3514.80.2.206 11220441

[B16] Hagger M. S. Wood C. Stiff C. Chatzisarantis N. L. (2009). The strength model of self-regulation failure and health-related behaviour. Health Psychol. Rev. 3, 208–238. 10.1080/17437190903414387

[B17] Haskell W. L. Lee I. M. Pate R. R. Powell K. E. Blair S. N. Franklin B. A.. (2007). Physical activity and public health: updated recommendation for adults from the American College of Sports Medicine and the American Heart Association. Med. Sci. Sports Exerc. 39, 1423–1434. 10.1249/mss.0b013e3180616b27 17762377

[B18] Hayes A. F (2012). PROCESS: A Versatile Computational Tool for Observed Variable Mediation, Moderation, and Conditional Process Modeling (White paper). Available online at: http://www.afhayes.com/public/process2012.pdf

[B19] Hofmann W. Baumeister R. F. Förster G. Vohs K. D. (2012). Everyday temptations: an experience sampling study of desire, conflict, and self-control. J. Pers. Soc. Psychol. 102, 1318–1335. 10.1037/a0026545 22149456

[B20] Hofmann W. Luhmann M. Fisher R. R. Vohs K. D. Baumeister R. F. (2014). Yes, but are they happy? Effects of trait self-control on affective well-being and life satisfaction. J. Person. 82, 265–277. 10.1111/jopy.12050 23750741

[B21] Lally P. Wardle J. Gardner B. (2011). Experiences of habit formation: a qualitative study. Psychol. Health Med. 16, 484–489. 10.1080/13548506.2011.555774 21749245

[B22] MacKinnon D. P. Fairchild A. (2009). Current directions in mediation analysis. Curr. Dir. Psychol. Sci. 18, 16–20. 10.1111/j.1467-8721.2009.01598.x 20157637 PMC2821103

[B23] Moffitt T. E. Arseneault L. Belsky D. Dickson N. Hancox R. J. Harrington H.. (2011). A gradient of childhood self-control predicts health, wealth, and public safety. Proc. Natl. Acad. Sci. U.S.A. 108, 2693–2698. 10.1073/pnas.1010076108 21262822 PMC3041102

[B24] Norcross J. C. Mrykalo M. S. Blagys M. D. (2002). Auld lang Syne: Success predictors, change processes, and self-reported outcomes of New Year's resolvers and nonresolvers. J. Clin. Psychol. 58, 397–405. 10.1002/jclp.1151 11920693

[B25] Orbell S. Verplanken B. (2010). The automaticity component of habit in health behavior: habit as cue-contingent automaticity. Health Psychol. 29, 374–383. 10.1037/a0019596 20658824

[B26] Phillips L. A. Gardner B. (2016). Habitual exercise instigation (vs. execution) predicts healthy adults' exercise frequency. Health Psychol. 35, 69. 10.1037/hea0000249 26148187

[B27] Ross R. Freeman J. A. Janssen I. (2000). Exercise alone is an effective strategy for reducing obesity and related comorbidities. Exerc. Sport Sci. Rev. 28, 165–170. 11064850

[B28] Sniehotta F. F. Scholz U. Schwarzer R. (2005). Bridging the intention-behaviour gap: planning, self-efficacy, and action control in the adoption and maintenance of physical exercise. Psychol. Health 20, 143–160. 10.1080/08870440512331317670

[B29] Tangney J. P. Baumeister R. F. Boone A. L. (2004). High self-control predicts good adjustment, less pathology, better grades, and interpersonal success. J. Pers. 72, 271–324. 10.1111/j/0022-3506.2004.00263.x 15016066

[B30] Verhoeven A. A. C. Adriaanse M. A. De Vet E. Fennis B. M. De Ridder D. T. D. (2014). Identifying the ‘if’ for ‘if-then’ plans: combining implementation intentions with cue-monitoring targeting unhealthy snacking behaviour. Psychol. Health 29, 1476–1492. 10.1080/08870446.2014.950658 25099386

[B31] Verplanken B. (2006). Beyond frequency: habit as a mental construct. Br. J. Soc. Psychol. 45, 639–656. 10.1348/014466605X49122 16984725

[B32] Verplanken B. Aarts H. (1999). Habit, attitude, and planned behaviour: is habit an empty construct or an interesting case of goal-directed automaticity? Eur. Rev. Soc. Psychol. 10, 101–134. 10.1080/14792779943000035

[B33] Verplanken B. Orbell S. (2003). Reflections on past behavior: a self-report index of habit strength. J. Appl. Soc. Psychol. 33, 1313–1330. 10.1111/j.1559-1816.2003.tb01951.x

[B34] Williams P. T. (2001). Physical fitness and activity as separate heart disease risk factors: a meta-analysis. Med. Sci. Sports Exerc. 33, 754–761. 10.1037/a0019596 11323544 PMC2821586

[B35] Wills T. A. Isasi C. R. Mendoza D. Ainette M. G. (2007). Self-control constructs related to measures of dietary intake and physical activity in adolescents. J. Adolesc. Health 41, 551–558. 10.1016/j/jadohealth.2007.06.013 18023783 PMC2190087

[B36] WMO (2012). Central Committee on Research Involving Human Subjects. Available online at: http://www.ccmo-online.nl

[B37] World Health Organization (2015). Physical Activity. Available online at: http://www.who.int/topics/physical_activity/en/

